# Genetics of serum urate concentrations and gout in a high-risk population, patients with chronic kidney disease

**DOI:** 10.1038/s41598-018-31282-z

**Published:** 2018-09-04

**Authors:** Jiaojiao Jing, Arif B. Ekici, Thomas Sitter, Kai-Uwe Eckardt, Elke Schaeffner, Yong Li, Florian Kronenberg, Anna Köttgen, Ulla T. Schultheiss

**Affiliations:** 1 Institute of Genetic Epidemiology, Department of Biometry, Epidemiology and Medical Bioinformatics, Medical Center–University of Freiburg, Faculty of Medicine, Freiburg, Germany; 2 Faculty of Biology, University of Freiburg, Freiburg, Germany; 3 Institute of Human Genetics, University of Erlangen-Nürnberg, Erlangen, Germany; 4 Department of Nephrology and Hypertension, Ludwig-Maximilians University, Munich, Germany; 5 Department of Nephrology and Medical Intensive Care, Charité, University-Medicine, Berlin, Germany; 6 Institute of Public Health, Charité, University-Medicine, Berlin, Germany; 7 Division of Genetic Epidemiology, Department of Medical Genetics, Molecular and Clinical Pharmacology, Innsbruck Medical University, Innsbruck, Austria; 8 Renal Division, Department of Medicine IV, Medical Center - University of Freiburg, Faculty of Medicine, Freiburg, Germany

## Abstract

We evaluated genetics of hyperuricemia and gout, their interaction with kidney function and medication intake in chronic kidney disease (CKD) patients. Genome-wide association studies (GWAS) of urate and gout were performed in 4941 CKD patients in the German Chronic Kidney Disease (GCKD) study. Effect estimates of 26 known urate-associated population-based single nucleotide polymorphisms (SNPs) were examined. Interactions of urate-associated variants with urate-altering medications and clinical characteristics of gout were evaluated. Genome-wide significant associations with serum urate and gout were identified for known loci at *SLC2A9* and *ABCG2*, but not for novel loci. Effects of the 26 known SNPs were of similar magnitude in CKD patients compared to population-based individuals, except for SNPs at *ABCG2* that showed greater effects in CKD. Gene-medication interactions were not significant when accounting for multiple testing. Associations with gout in specific joints were significant for *SLC2A9* rs12498742 in wrists and midfoot joints. Known genetic variants in *SLC2A9* and *ABCG2* were associated with urate and gout in a CKD cohort, with effect sizes for *ABCG2* significantly greater in CKD compared to the general population. CKD patients are at high risk of gout due to reduced kidney function, diuretics intake and genetic predisposition, making treatment to target challenging.

## Introduction

Gout is a progressive painful debilitating disease, and the most common inflammatory arthritis in many Western countries^1^. Population-based studies have identified genetic variants in multiple genes^2^ including *SLC2A9*^3–5^ and *ABCG2*^5,6^ associated with serum urate concentrations. Individuals with chronic kidney disease (CKD) represent a high-risk population for hyperuricemia and gout due to decreased renal clearance of urate and consecutive increase in serum urate concentrations. About ~25% of CKD patients from the prospective German Chronic Kidney Disease (GCKD) study reported a physician diagnosis of gout at study baseline and two thirds of patients were hyperuricemic^7^. This high prevalence of gout is most relevant given that CKD affects about 10% of the adult population in many countries^8^.

Despite the importance of CKD as a risk factor for gout, knowledge about genetic determinants of serum urate in the setting of CKD is limited. One candidate gene study that focused on 11 urate transporters reported that the strength of association - as quantified by the association p-value - between genetic variants in *ABCG2* was stronger in patients with CKD compared to 481 population-based individuals, while the opposite was observed for variants in *SLC2A9*^9^. While the authors hypothesized that this could be interpreted by a compensatory role of the ABCG2 transporter in the intestine in the setting of reduced kidney function, they did not formally compare effect sizes or test for differences in effect. Additional aspects of urate genetics in CKD that have not been addressed are interactions between genetic risk variants for gout and medication intake as well as clinical characteristics of gout attacks.

We aimed to evaluate the genetic underpinnings of hyperuricemia and gout in a large cohort of CKD patients, by carrying out genome-wide association studies (GWAS) of serum urate concentrations and gout. Effect sizes of known urate-associated variants detected in the general population were formally compared to their counterparts among CKD patients. Interactions between medications influencing serum urate concentrations and commonly prescribed in CKD were evaluated, and associations between genetic risk variants and clinical characteristics of gout were examined.

## Results

### Study population

Baseline clinical characteristics of 4,941 GCKD study participants with complete clinical information required for the GWAS are summarized in Table 1. A quarter of the patients reported diagnosis of gout at study baseline. Participants with gout compared to those without gout were significantly (p-value < 0.05) more likely to be male, older, have a higher BMI, be hypertensive, diabetic, have higher triglyceride levels and have lower eGFR. There were no significant differences in serum urate concentrations between the two groups, but patients with gout were significantly more likely to be treated with allopurinol compared to those without gout^7^. The most pronounced significant differences were observed for eGFR, age, gender, BMI, and diuretic and gout medication intake.

**Table 1:** Study sample characteristics of GCKD participants with complete urate, gout and genotype information.

Characteristic	Overall (n = 4941)	No gout (n = 3724)	Gout (n = 1217)	P-value
eGFR, ml/min/1.73 m^2^	49.5 ± 18.1	51.0 ± 18.9	44.9 ± 14.8	<2.2e-16
Age	60.1 ± 12.0	59.1 ± 12.6	63.1 ± 9.1	<2.2e-16
Female, n (%)	39.8%	44.5%	25.4%	<2.2e-16
BMI, kg/m^2^	29.8 ± 6.0	29.3 ± 5.8	31.6 ± 6.1	<2.2e-16
Hypertension, n (%)	96.2%	95.4%	98.6%	4.9E-07
Systolic BP, mmHg	139.4 ± 20.4	138.8 ± 20.1	141.2 ± 21.1	5.2E-04
Cholesterol, mg/dL	211.3 ± 53.0	213.0 ± 53.3	206.1 ± 51.8	6.0E-05
Triglycerides, mg/dL	168.1 (118.2, 239.0)	161.2 (113.8, 229.3)	193.6 (133.4, 270.4)	6.3E-15
CHD, n (%)	19.9%	17.5%	27.0%	6.4E-13
DM 2, n (%)	24.3%	21.8%	32.1%	4.3E-13
Serum urate, mg/dL	7.21 ± 1.92	7.20 ± 1.91	7.23 ± 1.96	7.3E-01
Hyperuricemia, n (%)	61.0%	57.2%	62.3%	1.8E-03
Alcohol intake, moderate to large amount	19.0%	17.3%	24.1%	9.4E-07
Diuretic intake, n (%)	61.0%	56.9%	73.7%	<2.2e-16
Gout medication intake, n (%)	32.8%	20.8%	69.5%	<2.2e-16

Continuous variables are mean (SD) unless otherwise noted. N = 4941, except for systolic blood pressure (n = 4913), cholesterol (n = 4935), triglycerides (n = 4933). Estimated glomerular filtration rate (eGFR), body mass index (BMI), systolic blood pressure (systolic BP), coronary heart disease (CHD), diabetes mellitus type 2 (DM 2); p-value for comparison of each characteristic in individuals with and without gout.

### Genome-wide association analyses

**Figure 1:**
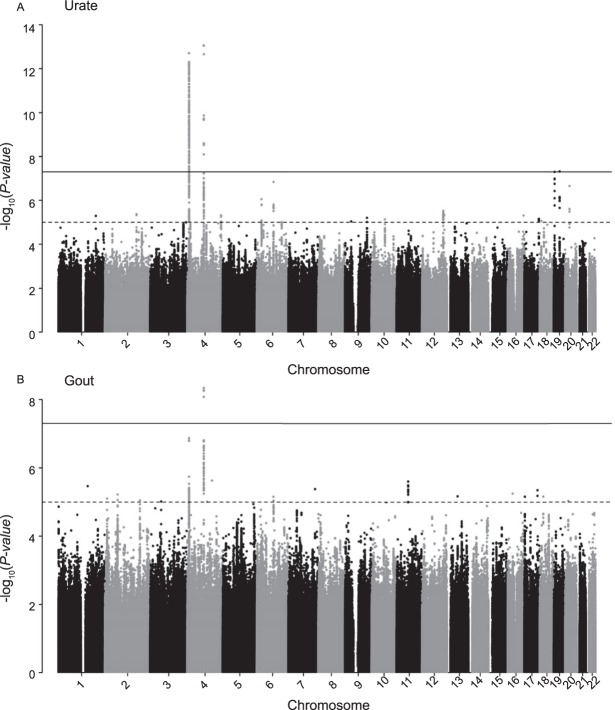
Manhattan plot for GWAS of serum urate (**A**) and gout (**B**) from the regression models. Y-axis: association p-values; x-axis: plotted SNPs by chromosomal position. Continuous line: genome-wide significance level (5E-8). Dashed line: suggestive significance level (1E-5). Data from 1000 Genomes imputation-based GWAS in CKD subjects of European-ancestry.

We conducted GWAS for serum urate concentrations and gout using 9,281,895 autosomal SNPs. A quantile-quantile plot comparing observed and expected p-values from the serum urate GWAS showed SNPs with very low observed p-values for serum urate and gout. There was no inflation of median test statistics, consistent with the absence of systematic errors (Supplemental Fig. 1). In the serum urate GWAS, three loci showed genome-wide significant associations (p-value < 5.0E-08, Fig. 1). Variants with the lowest association p-values mapped into the previously reported genes *SLC2A9* (rs13111638) and *ABCG2* (rs2231142). Per each minor allele copy, serum urate concentrations were 0.40 mg/dl (23.79 μmol/L) higher for *ABCG2* rs2231142 and 0.31 mg/dl (18.44 μmol/L) lower for *SLC2A9* rs13111638 (Table 2). A third locus, *URI1*, had not been detected in prior population-based studies. Because CKD cohorts with gout information were not available for replication, and because our findings supported the presence of similar associated loci in population-based and CKD patient studies, the index SNP at this locus was investigated in the well-powered summary results from GWAS meta-analysis of serum urate of >140,000 participants in the GUGC Consortium^2^. The association was not significant and therefore not followed up on. Odds Ratios (OR) for the associations with gout from the GWAS of gout were 1.54 (95% CI: 1.34; 1.78) for *ABCG2* rs2231142 and 0.77 (95% CI: 0.68; 0.86) for *SLC2A9* rs13111638 (Table 2). Leading SNPs with association p-values of p < 1.0E-06 (suggestive significance) are listed in Supplemental Tables 1 and 2.

**Table 2:** Association estimates for index SNPs associated at genome-wide significance with serum urate, and their corresponding association with gout.

SNP	Funct.	Closest Genes	Chr	Position	A1	A2	Freq.A1	Urate			Gout		
								effect	SE	p-value	OR	95% CI	p-value
rs2231142	exonic	*ABCG2*	4	89052323	T	G	0.11	0.40	0.05	8.8E-14	1.54	1.34, 1.78	5.3E-09
rs13111638	intronic	*SLC2A9*	4	9996890	T	C	0.19	−0.31	0.04	2.0E-13	0.77	0.68, 0.86	2.9E-05

SNP indicates the SNP with the lowest p-value for urate; the index SNP at *SLC2A9* for gout was rs59834205 (p-value 1.36E-07, LD with rs13111638: r^2^ = 0.60, D′ = 0.79, using 1000 G phase 3); the index SNP at *ABCG2* for gout was rs4148155 (p-value 4.54E-09; LD with rs2231142: r^2^ = 0.99, D′ = 1, using 1000 G phase 3). Estimates for rs4148155 and rs59834205 are shown in Supplemental Table 2. Abbreviations: Funct., function; Chr, chromosome; A1, coded allele; A2, non-coded allele; Freq.A1, the frequency of the coded allele; effect, effect of A1; SE, standard error; OR, odds ratio; 95% CI, 95% confidence interval. Associations with urate were adjusted for age, sex, log(eGFR), BMI, significant principal components (p < 0.05), intake of diuretics and gout medication, while associations with gout were adjusted for age, sex, log(eGFR), BMI, and intake of diuretics. Associations were filtered for plausibility and quality.

### Comparison of genetic effects to the general population

**Figure 2:**
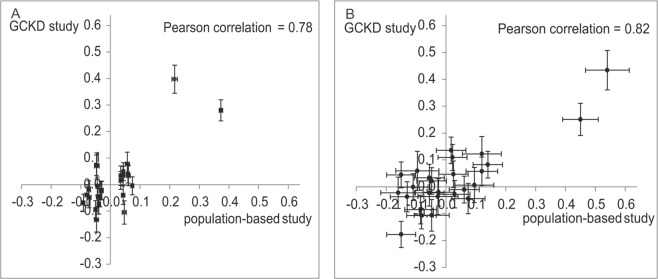
Scatter plot comparing effect sizes of 26 known urate-associated SNPs from a meta-analysis of population-based studies (X-axis) to those of CKD patients (Y-axis). Upper panel shows the association with urate (Panel A), lower panel shows the associations with gout (Panel B). Gout effects are presented on the log odds scale.

Next we compared the effect sizes of 26 SNPs known to be associated with serum urate in the general population^2^ to the results from the GWAS in the GCKD study. Of the 26 SNPs discovered among approximately 140,000 individuals, seven SNPs were nominally associated (p-value < 0.05) with serum urate among CKD patients in the GCKD study (Supplemental Table 3) and two SNPs, at *ABCG2* and at *SLC2A9*, were significant after adjusting for multiple testing (p-value < 1.9E-03, 0.05/26). Effect sizes of the genetic associations on serum urate were of similar magnitude in the GCKD study and the population-based meta-analysis for most SNPs, with high correlations across all risk variants observed for both urate (Pearson correlation coefficient 0.78 (Fig. 2, Panel A)) and gout (correlation 0.82 (Fig. 2, Panel B)). The two SNPs that were significantly associated in the GCKD study, at *SLC2A9* and *ABCG2*, were tested for differences in genetic effect sizes on serum urate. The association for rs2231142 in *ABCG2* was significantly higher in CKD patients compared to the results from the general population (effect 0.4 mg/dl in CKD vs. 0.22 mg/dl in population-based studies, p-value for difference = 1.5E-03; Supplemental Table 4).

### SNP-kidney-function interaction

Although genetic effects of most variants on serum urate discovered in the general population were similar among CKD patients, the effect of individual variants on serum urate may change as eGFR declines. We therefore compared associations of the two genome-wide significant urate-associated SNPs across different categories of eGFR. When stratified by eGFR, the minor T allele of rs2231142 in *ABCG2* was significantly associated with serum urate concentrations in each stratum and showed a trend for larger effects at eGFR values <30 ml/min/1.73 m^2^ (Supplemental Table 5). A statistical test for interaction across the entire eGFR range was not significant. The SNP in *SLC2A9* did not show any clear effect size differences or interactions with categories of eGFR.

### Gene-environment interaction of candidate variants and medications

Several medications commonly prescribed to CKD patients are known to affect serum urate concentrations. We therefore assessed potential effect modifications of these medications on the associations of urate-associated SNPs mapping into the interacting transport proteins with serum urate and gout. The intake of allopurinol, thiazide diuretics, loop diuretics, potassium sparing diuretics and losartan was evaluated for interaction with known urate-associated SNPs mapping into the loci for *SLC2A9*, *ABCG2*, *SLC17A1*, and *SLC22A12* (p < 2.5E-03, 0.05/(4 * 5)). Table 3 shows results for the interaction between all SNPs and medications that showed at least a nominally significant (p < 0.05) SNP* medication interaction, namely thiazides and loop diuretics. Two models were evaluated because of polypharmacy in patients of moderate and advanced CKD. One compared the effect between patients taking the respective medication to those not taking it, and one compared the effect between patients who were only taking this medication to patients not taking any of the evaluated medications. Three of the interactions achieved nominal significance in at least one comparison and had similar effect size differences in both types of comparisons, but none of these interactions were statistically significant after correcting for multiple testing. A detailed list of all tested gene-medication interactions can be found in Supplemental Table 6.

**Table 3:** Selected interactions between known urate-associated risk alleles in urate transporters and medications known to affect serum urate concentrations.

SNP	Medication	With this medication				Without this medication				All (SNP × medication)			
		effect	SE	p-value	n	effect	SE	p-value	n	effect	SE	p-value	n
*SLC2A9* rs12498742 (G)	thiazide	−0.23	0.08	2.7E-03	1570	−0.33	0.05	7.2E-13	3371	0.11	0.08	2.0E-01	4941
	loop diuretic	−0.37	0.07	1.0E-07	1900	−0.25	0.05	1.5E-07	3041	−0.08	0.08	2.9E-01	4941
*ABCG2* rs2231142 (T)	thiazide	0.37	0.10	3.1E-04	1570	0.43	0.06	3.0E-12	3371	−0.06	0.11	6.0E-01	4941
	loop diuretic	0.58	0.10	2.1E-09	1900	0.30	0.06	9.3E-07	3041	0.22	0.11	4.4E-02	4941
*SLC17A1* rs1165151 (G)	thiazide	0.14	0.06	2.5E-02	1570	0.03	0.04	4.4E-01	3371	0.10	0.07	1.5E-01	4941
	loop diuretic	−0.03	0.06	6.2E-01	1900	0.11	0.04	3.1E-03	3041	−0.16	0.07	2.0E-02	4941
*NRXN2*/*SLC22A12* rs478607 (A)	thiazide	−0.13	0.09	1.5E-01	1570	−0.12	0.05	2.1E-02	3371	−0.02	0.10	8.7E-01	4941
	loop diuretic	−0.29	0.08	4.5E-04	1900	−0.01	0.05	7.9E-01	3041	−0.27	0.09	3.6E-03	4941
**SNP**	**Medication**	**Only this medication**				No medication**				**All (SNP** × **medication)**			
		**effect**	**SE**	**p-value**	**n**	**effect**	**SE**	**p-value**	**n**	**effect**	**SE**	**p-value**	**n**
*SLC2A9* rs12498742 (G)	thiazide	−0.06	0.12	5.9E-01	604	−0.29	0.06	3.9E-06	1503	0.23	0.12	6.3E-02	2107
	loop diuretic	−0.44	0.11	6.3E-05	589	−0.29	0.06	3.9E-06	1503	−0.17	0.12	1.6E-01	2092
*ABCG2* rs2231142 (T)	thiazide	0.35	0.16	3.3E-02	604	0.27	0.09	1.9E-03	1503	0.11	0.17	5.1E-01	2107
	loop diuretic	0.66	0.17	1.6E-04	589	0.27	0.09	1.9E-03	1503	0.40	0.18	2.4E-02	2092
*SLC17A1* rs1165151 (G)	thiazide	0.24	0.10	2.1E-02	604	0.02	0.05	6.7E-01	1503	0.21	0.11	4.6E-02	2107
	loop diuretic	−0.02	0.10	8.8E-01	589	0.02	0.05	6.7E-01	1503	−0.06	0.10	5.9E-01	2092
*NRXN2*/*SLC22A12* rs478607 (A)	thiazide	0.04	0.14	7.5E-01	604	−0.03	0.07	6.9E-01	1503	0.08	0.14	5.8E-01	2107
	loop diuretic	−0.23	0.13	9.0E-02	589	−0.03	0.07	6.9E-01	1503	−0.21	0.14	1.4E-01	2092

**No medication refers to not taking gout medications, diuretics and losartan. Associations were adjusted for age, sex, log(eGFR), BMI, and medications (gout medication, diuretics, losartan) other than the category being evaluated. The p-value for interaction adjusted for multiple testing was p < 2.5E-03. Rs12498742 (*SLC2A9*) is the reported index SNP from the GUGC Consortium and is in LD with rs13111638 (r^2^ = 0.74, D′ = 0.98).

### Association with clinical characteristics of gout

Hyperuricemia can lead to monosodium urate crystal formation, influenced by factors such as temperature, pH, salt concentration, and cartilage matrix components. Processes of crystal formation and initiation of joint inflammation are incompletely understood and more underlying factors seem to be at play. We investigated whether genetic effects were more clearly discernible in joints across which temperature, weight pressure or usage strain may differ for the genome-wide significant urate-associated index SNPs at *ABCG2* and *SLC2A9*. The SNP rs12498742 in *SLC2A9* was significantly (p < 3.1E-03, 0.05/(2 * 8)) associated with gout affection in wrist and midfoot joints (Table 4). The strongest effect was observed with gout affection of the wrists (OR 0.35, 95% CI 0.18–0.60, p = 4.7E-04), with each copy of the G allele associated with 65% lower odds of gout affection of the wrists. Because of the lower minor allele frequency, subgroups for rs2231142 in *ABCG2* were small, and only one joint localization showed a nominally significant (p < 0.05) association (ankles, OR = 1.47, 95% CI 1.02–2.08, p = 3.2E-02). The possibility to study a clear pattern of joint affection in relation to genetic risk variants therefore requires study in even larger samples.

**Table 4:** Association between genome-wide significant SNPs and clinical characteristics of gout.

Affected joints	Controls	Cases	% of female cases	*SLC2A9* rs12498742 (G)		*ABCG2* rs2231142 (T)	
				OR (95%CI)	p-value	OR (95%CI)	p-value
shoulder	2007	19	26.3%	0.67 (0.25–1.47)	3.6E-01	0.38 (0.06–1.27)	1.9E-01
elbow	1988	38	10.5%	0.36 (0.15–0.73)	9.7E-03	0.92 (0.42–1.77)	8.2E-01
wrist	1959	67	23.9%	0.35 (0.18–0.60)	**4**.**7E-04**	1.18 (0.70–1.89)	5.1E-01
finger	1929	97	35.1%	0.73 (0.49–1.04)	9.7E-02	0.64 (0.37–1.04)	9.3E-02
knee	1928	98	7.1%	0.59 (0.39–0.87)	1.0E-02	1.41 (0.94–2.07)	8.7E-02
ankle	1903	123	14.6%	0.67 (0.47–0.93)	2.3E-02	1.47 (1.02–2.08)	3.2E-02
toe	1504	522	20.5%	0.92 (0.77–1.09)	3.3E-01	1.15 (0.93–1.43)	1.9E-01
midfoot	1816	210	21.4%	0.61 (0.46–0.80)	**4**.**2E-04**	1.31 (0.97–1.73)	7.0E-02

Covariates included age, sex and log (eGFR). OR: Odds Ratio, 95% CI: 95% Confidence interval. For *SLC2A9*, rs12498742 is the previously reported index SNP from the GUGC Consortium; it is in LD with rs13111638 (r^2^ = 0.74, D′ = 0.98), the index SNP for *SLC2A9* in the GCKD study. The p-value adjusted for multiple testing was p < 3.1E-03 (in bold).

## Discussion

In this study of serum urate and gout genetics in CKD, we found genome-wide significant associations with SNPs in two previously identified loci, *ABCG2* and *SLC2A9*. The magnitude of genetic effects identified in previous population-based studies was generally similar except for *ABCG2*, where effects were larger in CKD patients. Effect modification of risk variants in *ABCG2* and *SLC2A9* by eGFR categories or intake of urate-altering medications was not significant after accounting for multiple testing, but there were several nominally significant gene-by-medication interactions. Genetic associations with detailed information about the affected joint localization showed significant associations for the *SLC2A9* risk allele with gout of the wrists and midfoot joints.

*SLC2A9* and *ABCG2* are the two most prominent players in gout explaining ~5% of variance in serum urate levels^10^. GLUT9, affecting uric acid transport through urate reabsorption at the basolateral membrane^11^ of renal proximal tubules, is encoded by *SLC2A9*. The ABCG2 transporter, encoded by *ABCG2* and localized to the brush border of renal proximal tubules and intestinal cells, functions to extrude urate in an ATP-dependent fashion^2,5,12–18^.

Similar effect sizes of the lead SNP in *SLC2A9* were found when comparing our results to those from population-based studies^2,4,5^ and one previous study in individuals with CKD stage 3. The previous study of CKD patients by Bhatnagar and colleagues evaluated a bi-racial study population with fewer and younger patients overall and smaller patient numbers in the European ancestry subgroup^9,19^. Other differences include a genome-wide screen in our study, allowing for the comparison of effect sizes outside of the 11 transporter genes they studied, and the detailed clinical characterization of gout in our study. While Bhatnagar and colleagues^9^ reported that the index variant in *ABCG2* in CKD patients was associated with serum urate with similar effect sizes but at lower p-values when compared to population-based studies^2,4,5^, we found that the effect sizes were almost twice as large in CKD patients compared to population-based samples. Differences between the studies, most importantly differences in ethnic composition and the proportion of individuals with diabetes, but also in age may account for this difference, but random variation should also be taken into consideration.

Besides renal excretion, recent findings demonstrated physiological and pathophysiological roles of *ABCG2* on intestinal urate excretion in humans^9,20^, where the degree of intestinal *ABCG2* dysfunction showed strong associations with the severity of hyperuricemia in the setting of end-stage renal disease^20^. We further tested whether the risk conferred by rs2231142 in *ABCG2* was altered in the setting of reduced eGFR using stratified and interaction analyses. A trend for higher genetic effects on serum urate levels in individuals with eGFR <30 ml/min/1.73 m^2^ lends some support to the importance of intestinal ABCG2 function, but results from our interaction analysis with eGFR did not support the hypothesis that genetic effects of *ABCG2* on serum urate become larger i.e. more important in individuals with CKD compared to the general population in a linear fashion.

To our knowledge, this is the first study to quantify the interaction between gout risk genes and sub-classes of diuretics or allopurinol. Diuretics are widely used for CKD management, hypertension and heart failure^21^, the latter being frequent comorbidities in patients of the GCKD study. Allopurinol is the most frequently prescribed urate-lowering therapy in the GCKD study despite its side effects and a reduced dose recommendation in CKD. Our findings are suggestive of a greater impact of *ABCG2* risk alleles among those using loop diuretics. The observation that adjustment for allopurinol intake was important in detecting genetic effects on gout in this study implies that allopurinol is a well working drug regardless of the observed *SLC2A9* and *ABCG2* genotype.

Gene-environment interaction-studies have shown conflicting results for the role of genetic risk factors in gout patients using diuretics^22–24^, likely related to polypharmacy in CKD and limited sample sizes. Such sample sizes have to be several times larger than the ones needed to detect main gene effects, highlighting the need for future, bigger cohort studies to explore gene-environment interactions. Worldwide rates of initiation and continuation of urate-lowering therapy are low and achievement of serum urate targets therefore infrequent^7^, although gout in combination with CKD^7^ contributes to poor health-related quality of life in these patients^25^.

Our results on joint location of acute gout flares do not clearly answer the hypothesis that genetic effects are more important in joints with higher weight pressure and usage strain^26^. However, sample sizes of each subgroup were small, highlighting the need of large gout research cohorts.

Strengths of our study are being one of the largest CKD cohorts world-wide, a detailed data collection, including medication intake data and information from a validated gout questionnaire^27–29^ based on ACR criteria to survey gout, the availability of genome-wide genetic data for all patients allowing for an unbiased search for gout risk loci in the setting of CKD and a comprehensive examination of known genetic risk loci. Knowledge generated from CKD cohorts such as the GCKD study may inform future interventional trials that focus on reducing the burden of hyperuricemia and its associated progression of CKD and CVD^30–34^.

The present study has some limitations. Gout status was based on self-reported physician diagnosis of gout, possibly introducing some misclassification, but good sensitivity and reliability on self-reported gout has been reported previously^7,35^. Our analyses were based on a Caucasian patient population of mostly CKD stage 3, potentially compromising generalizability. Sample sizes were small for interaction analyses, although the GCKD study is one of the largest observational cohorts of CKD patients world-wide. Pooled samples from different CKD cohorts with available information on serum urate concentrations and gout under the framework of CKD consortia around the world could address this limitation^36^.

In summary, the known genetic associations at *ABCG2* and *SLC2A9* with serum urate and gout were replicated in CKD patients with higher effect sizes for *ABCG2* in patients with CKD compared to individuals from the general population. Interactions with medications and effect modification by eGFR categories were not significant after correcting for multiple testing. Affected joint localizations showed significant associations for *SLC2A9* with wrists and midfoot joints. Patients with CKD are at risk for gout due to reduced kidney function, diuretics intake and genetics^7^. This large and complex patient group will become even more relevant in ageing populations and with increasing polypharmacy, and yet is still understudied.

## Methods

### Study population

The GCKD study is a multi-centric, prospective observational study of 5,217 CKD patients of European ancestry. Patients undergo regular, standardized study visits including questionnaires, physical examinations and biosampling by trained personnel. The GCKD study has implemented a data protection concept according to the data protection recommendations of the platform for technology and methods for networked medical research (TMF; www.tmf-ev.de), supported by the German Ministry of Education and Research (www.bmbf.de). The study protocol was approved by local institutional review boards (1 ethics committee of the RWTH Aachen University, Aachen, Germany; 2 ethics committee of the Charité – University-Medicine, Berlin, Germany; 3 ethics committee of the Friedrich-Alexander University, Erlangen, Germany; 4 ethics committee of the Albert-Ludwigs-University, Freiburg, Germany; 5 ethics committee of the Friedrich-Schiller University, Jena, Germany; 6 ethics committee of the Hannover Medical School, Hannover, Germany; 7 ethics committee of the medical faculty, Ruprecht-Karls University, Heidelberg, Germany; 8 ethics committee of the medical faculty, Ludwig-Maximilians-University, Munich, Germany; 9 ethics committee of the Julius-Maximilians-University, Würzburg, Germany) at each participating academic institution, the data protection concept was reviewed by the data protection officer of the State of Hessen^37^ and the study was registered in the national registry of clinical studies (DRKS 0003971). All methods were carried out in accordance with relevant guidelines and regulations. Written informed consent was obtained for all participants. Study procedures and main baseline findings have been reported^19,37^. Prevalence and correlates of gout at study baseline were published elsewhere^7^.

A comprehensive gout questionnaire, implemented due to the high prevalence of gout observed at study baseline, designed to meet the American College of Rheumatology (ACR) criteria used to survey gout and validated in previous observational studies of gout^27–29^, was used to assess clinical characteristics of patients, reporting a physician diagnosis of gout. The questionnaire covered physician diagnosis of gout, age of onset, annual number of attacks, attack duration, appearance of the affected joints, affected joint number and localization, presence of tophi, presence of crystals from fine-needle aspiration, presence of hyperuricemia, and family history of gout.

### Genotyping, quality control and imputation

DNA was isolated from peripheral blood leukocytes and genotyped for 5,123 patients using the Illumina HumanOmni2.5-8 v1.2 BeadChip (Illumina, GenomeStudio, Genotyping Module Version 1.9.4) at the Helmholtz Center Munich. Standardized protocols were used for data cleaning^38^. Custom written scripts (R, Perl) and plink1.9^39^ software was used for quality control. Quality control steps included call rate, sex check, mean heterozygosity, genetic ancestry and cryptic relatedness, leading to the exclusion of 89 samples. Single nucleotide polymorphism (SNP) exclusion criteria prior to imputation were a call rate of <0.96, deviation from Hardy-Weinberg equilibrium (p < 1.0E-05), and duplicate positions, yielding clean genotype data for 2,337,794 autosomal SNPs of the Omni 2.5 component from 5,034 patients. Genotypes were then imputed by pre-phasing^40^ and imputation with Impute2 (v2.3.1)^41^ using the 1000 Genomes Phase 3 version 5 ALL as reference panel. After genotype imputation, 9,281,895 SNPs of high imputation quality (info score > 0.8) and a minor allele frequency of >0.01 were kept for association analyses to generate robust association statistics.

### Statistical analyses

Patient characteristics were compared using t-tests for continuous and Chi-square tests for categorical variables.

#### Genome-wide association analyses

Single variant genome-wide association analyses were conducted using linear regression (urate) and logistic regression (gout), assuming an additive genetic model using genotype dosages to account for imputation uncertainty. For urate as the outcome, the regression model was adjusted for age, sex, estimated glomerular filtration rate (log(eGFR)), body mass index (BMI), intake of diuretics, and intake of gout medication. For gout as the outcome, the models was adjusted for age, sex, log(eGFR), BMI, and intake of diuretics. In addition, principal components (PCs) were included as covariates when significantly (p < 0.05) associated with evaluated outcomes of the respective models.

Association analyses were performed using SNPTEST v2.5^42^. Functional annotation of variants was conducted using ANNOVAR^43^.

#### Comparison of genetic effects to the general population

Twenty-six SNPs previously reported as associated with serum urate and replicated in a meta-analysis of mostly population-based cohorts in the Global Urate Genetics Consortium (GUGC)^2^ were used as candidate variants comparing their effect estimates in general populations with CKD patients in the GCKD study. Differences in effect estimates were computed for significantly associated SNPs (in the GCKD study) after adjusting for multiple testing (p-value < 1.9E-03, 0.05/26) between population-based results and CKD patients using a 2-sample t-test. SNP associations were compared for minor allele frequencies, direction and magnitude and evaluated visually.

#### SNP-kidney-function interaction

Interactions were evaluated for SNPs showing genome-wide significant associations with serum urate in the GCKD study. According to the Kidney Disease: Improving Global Outcomes CKD guidelines^44^, eGFR was categorized into stages G1/2 (≥60 ml/min/1.73 m^2^), G3a (45–59 ml/min/1.73 m^2^), G3b (30–44 ml/min/1.73 m^2^) and G4/5 (˂30 ml/min/1.73 m^2^). For each of the four eGFR categories, the multivariable adjusted odds ratios (OR) and 95% confidence intervals (95% CI; all covariates as above except for eGFR) were compared. Interactions between genotype and eGFR category were evaluated by adding a SNP*eGFR category term to the model and evaluating its statistical significance.

#### Gene-environment interaction of candidate variants and medications

Gene environment-interactions were assessed for known urate-associated variants^2^ mapping into or near transporter genes reported to interact with medications (*SLC2A9*, *ABCG2*, *NRXN2*/*SLC22A12* and *SLC17A1*)^11^ and medications known to affect urate concentration and commonly prescribed in CKD patients: allopurinol, thiazide diuretics, loop diuretics, potassium sparing diuretics and losartan. Allele dosages of imputed candidate SNPs were converted to the most likely genotypes. Current medication intake was assessed for all GCKD patients and coded using Anatomical Therapeutic Chemical (ATC) codes. Allopurinol, thiazide diuretics, loop diuretics, potassium sparing diuretics and losartan intake were represented by codes M04AA01/M04AA51, C03A/C03B C03C, C03D and C09CA01.

For each SNP and medication combination, two types of stratified and interaction analyses were performed adjusted for age, sex, log(eGFR), BMI, intake of medications other than the evaluated class and PCs significantly associated with serum urate. Firstly, the stratified analyses by intake of the specific medication under evaluation and the analysis including an interaction term (SNP × specific medication) were assessed among all individuals. Secondly, the stratified analyses by intake of the specific medication under evaluation and the analysis including an interaction term (SNP × specific medication) were assessed excluding individuals taking any of the other classes of medication under evaluation. Statistical significance for multiple testing (p < 2.5E-03) was obtained by dividing the significance threshold of 0.05 by the four tested SNPs multiplied with the five evaluated medications.

#### Association with clinical characteristics of gout

SNPs associated with serum urate at genome-wide significance were characterized for their associations with clinical characteristics of gout. Case numbers were only sufficient for a comparison of affected joint localizations across genotypes. Because of the limited sample size, only the most important covariates, age, sex and log(eGFR), were included. Statistical significance for multiple testing (p < 3.1E-03) was obtained by dividing the significance threshold of 0.05 by the two tested SNPs multiplied with the eight evaluated locations of gout attacks. All candidate variant analyses were conducted using R version 3.3.0.

## Electronic supplementary material

Supplemental Information

## Data Availability

The data that supports the findings of this study is available for authors upon reasonable request and with permission of the Steering Committee of the GCKD study http://www.gckd.org.
