# Supplementary material for: Genetics of serum urate concentrations and gout in a high-risk population, patients with chronic kidney disease
Source: Sci Rep. 2018 Sep 4;8:13184. doi: 10.1038/s41598-018-31282-z (PMC6123425; doi:10.1038/s41598-018-31282-z)
Supplement: Supplementary file 1 — Supplemental Information [file 41598_2018_31282_MOESM1_ESM.pdf]

Genetics of serum urate concentrations and gout in a high-risk population, patients with chronic kidney disease

SUPPLEMENTAL INFORMATION

Jiaojiao Jing[1, 2], Arif B. Ekici[3], Thomas Sitter[4], Kai-Uwe Eckardt[5], Elke Schaeffner[6], Yong Li[1], Florian Kronenberg[7], Anna Köttgen[1, *], Ulla T. Schultheiss[1, 8]

Affiliations:
[1]Institute of Genetic Epidemiology, Department of Biometry, Epidemiology and Medical Bioinformatics, Medical Center – University of Freiburg, Faculty of Medicine, Freiburg, Germany
[2]Faculty of Biology, University of Freiburg, Freiburg, Germany
[3]Institute of Human Genetics, University of Erlangen-Nürnberg, Erlangen, Germany
[4]Department of Nephrology and Hypertension, Ludwig-Maximilians University, Munich, Germany
[5]Department of Nephrology and Medical Intensive Care, Charité, University-Medicine, Berlin, Germany
[6]Institute of Public Health, Charité, University-Medicine, Berlin, Germany
[7]Division of Genetic Epidemiology, Department of Medical Genetics, Molecular and Clinical Pharmacology, Innsbruck Medical University, Innsbruck, Austria
[8]Renal Division, Department of Medicine IV, Medical Center - University of Freiburg, Faculty of Medicine, Freiburg, Germany

Table of Contents

SUPPLEMENTAL FIGURES……………………………………………………………3

SUPPLEMENTAL FIGURE 1: QQ PLOTS FOR URATE (A) AND GOUT (B)......................................3

SUPPLEMENTAL TABLES...4-10

SUPPLEMENTAL TABLE 1: INDEPENDENT INDEX SNPS ASSOCIATED WITH SERUM URATE CONCENTRATIONS FOR P-VALUES <1E-6. 4

SUPPLEMENTAL TABLE 2: INDEPENDENT INDEX SNPS ASSOCIATED WITH GOUT FOR P-VALUES <1E-6. 6

SUPPLEMENTAL TABLE 3: ASSOCIATION OF 26 PREVIOUSLY REPORTED SNPS DISCOVERED AMONG ~ 140,000 INDIVIDUALS FROM THE GUGC CONSORTIUM IN THE GCKD STUDY COHORT (N=4941) FOR SERUM URATE CONCENTRATIONS. 6

SUPPLEMENTAL TABLE 4: EFFECT SIZE COMPARISON OF PREVIOUSLY REPORTED SNPS DISCOVERED AMONG ~ 140,000 INDIVIDUALS FROM THE GUGC CONSORTIUM AND SERUM URATE WITH GENOME-WIDE SIGNIFICANT SNPS IN THE GCKD STUDY COHORT (N=4941)...7

SUPPLEMENTAL TABLE 5: ASSOCIATION BETWEEN GENOME-WIDE SIGNIFICANT SNPS AND SERUM URATE ACROSS DIFFERENT CATEGORIES OF EGFR. 8

SUPPLEMENTAL TABLE 6: DETAILED GENE-MEDICATION INTERACTIONS FOR SELECTED SNPS AND ALLOPURINOL, THIAZIDES, LOOP DIURETICS, POTASSIUM SPARING DIURETICS OR LOSARTAN.. 9-10

Supplemental Figure 1: QQ plots for urate (**A**) and gout (**B**)

A

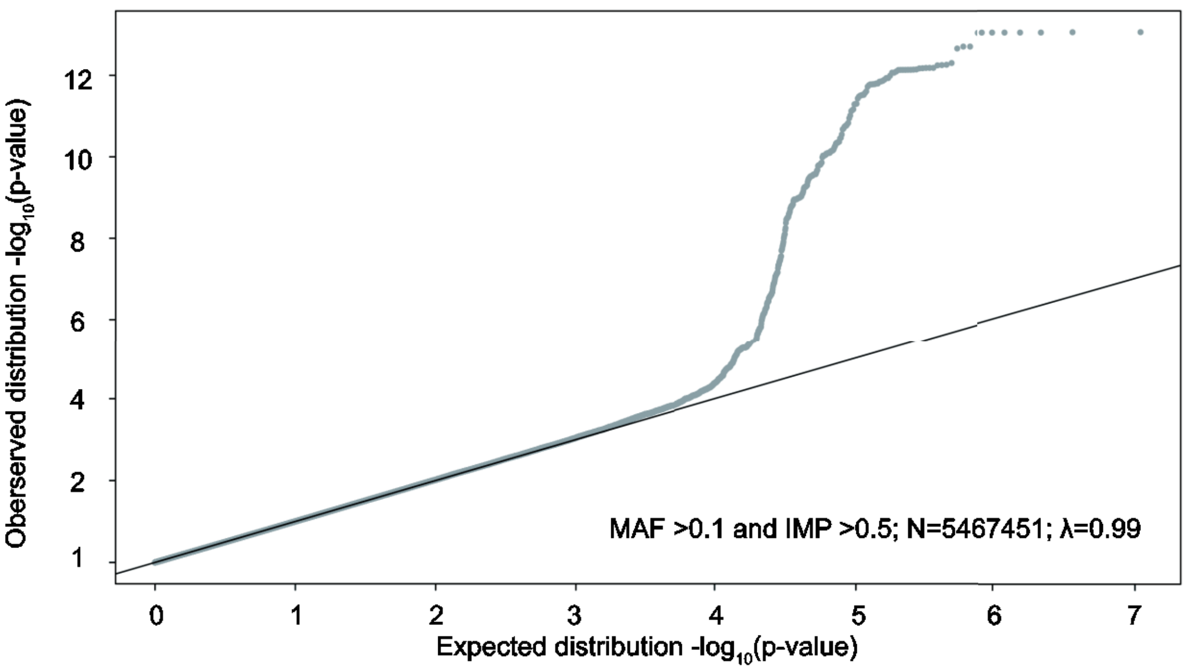

B

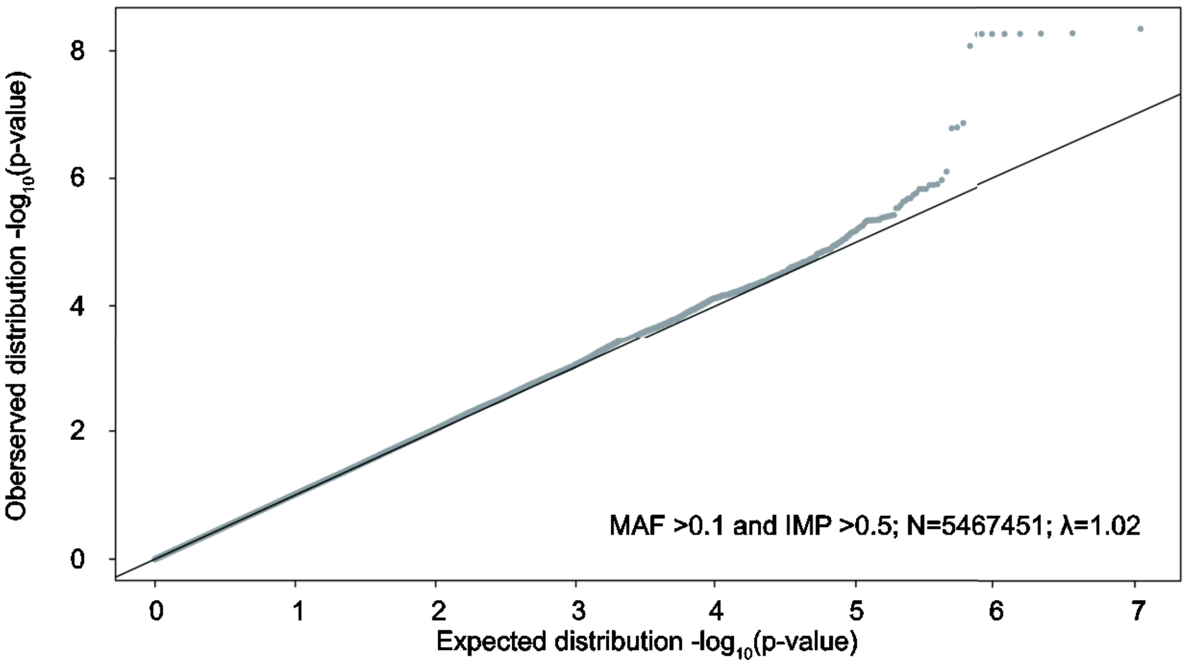

QQ plots: Quantile-Quantile plots; Urate adjusted for age, sex, log(eGFR), BMI, significant principal components, and intake of diuretics and gout medication. Gout: adjusted for age, sex, log(eGFR), BMI, intake of diuretics.

Supplemental Table 1: Independent index SNPs associated with serum urate concentrations at p-values <1E-6.

SNP	Closest gene	Chr	Position	A1	A2	Freq.A1	Effect	SE	p-value	n
rs13111638	*SLC2A9*	4	9996890	T	C	0.19	-0.31	0.043	2.0E-13	4941
rs2231142	*ABCG2*	4	89052323	T	G	0.11	0.40	0.053	8.8E-14	4941
rs9379832	*HIST1H2BE*	6	26186200	G	A	0.33	-0.18	0.036	8.9E-07	4941
rs62416056	*BACH2*	6	90627773	C	G	0.17	0.23	0.044	1.4E-07	4941
rs76909566	*NFIC*	19	3456656	A	G	0.09	0.33	0.060	5.0E-08	4941
rs4805497	*URI1*	19	30405685	A	G	0.92	0.34	0.061	4.8E-08	4941
rs78260152	*FLJ33581*	20	24126761	T	C	0.07	0.34	0.065	2.2E-07	4941

SNP: SNP with the lowest p-value; Chr: chromosome; A1: coded allele; A2: non-coded allele; Freq.A1: frequency of the coded allele; Effect: effect of A1; SE: standard error. Associations with urate were adjusted for age, sex, log(eGFR), BMI, significant principal components ($p<0.05$), and intake of diuretics and gout medication.

Supplemental Table 2: Independent index SNPs associated with gout at p-values <1.0E-06.

SNP	Closest gene	Chr	Position	A1	A2	Freq.A1	OR	95% CI	p-value	n
rs59834205	*SLC2A9*	4	9961019	CA	C	0.17	0.69	0.60, 0.79	1.4E-07	4941
rs4148155	*ABCG2*	4	89054667	G	A	0.11	1.55	1.34, 1.79	4.5E-09	4941

SNP: SNP with the lowest p-value; Chr: chromosome; A1: coded allele; A2: non-coded allele; Freq.A1: frequency of the coded allele; OR: odds ratio; 95% CI: 95% confidence interval. Associations with gout were adjusted for age, sex, log(eGFR), BMI, intake of diuretics.

Supplemental Table 3: Association of serum urate with 26 previously reported SNPs discovered among 140,000 individuals from the GUGC Consortium in the GCKD study cohort (N=4941)

SNP	Closest gene	Chr	Position	A1	A2	Freq.A1	effects	SE	P-value
rs1471633	*PDZK1*	1	145723739	C	A	0.53	-0.04	0.03	2.4E-01
rs1260326	*GCKR*	2	27730940	C	T	0.58	0.00	0.03	9.0E-01
rs12498742	*SLC2A9*	4	9944052	G	A	0.23	-0.28	0.04	**1.9E-12**
rs2231142	*ABCG2*	4	89052323	T	G	0.11	0.40	0.05	**8.8E-14**
rs675209	*RREB1*	6	7102084	C	T	0.74	-0.03	0.04	3.8E-01
rs1165151	*SLC17A1*	6	25821616	G	T	0.51	0.07	0.03	3.8E-02
rs1171614	*SLC16A9*	10	61469538	C	T	0.77	0.04	0.04	3.2E-01
rs2078267	*SLC22A11*	11	64334114	T	C	0.51	-0.02	0.03	5.6E-01
rs478607	*NRXN2*	11	64478063	A	G	0.84	-0.13	0.05	4.0E-03
rs3741414	*INHBC*	12	57844049	T	C	0.23	-0.05	0.04	2.3E-01
rs11264341	*TRIM46*	1	155151493	T	C	0.40	-0.09	0.03	5.2E-03
rs17050272	*INHBB*	2	121306440	A	G	0.43	0.02	0.03	6.5E-01
rs6770152	*SFMBT1*	3	53100214	T	G	0.59	-0.01	0.03	8.6E-01
rs17632159	*TMEM171*	5	72431482	C	G	0.31	-0.07	0.04	4.0E-02
rs729761	*VEGFA*	6	43804571	G	T	0.74	-0.07	0.04	5.8E-02
rs1178977	*BAZ1B*	7	72857049	G	A	0.17	0.11	0.04	1.7E-02
rs10480300	*PRKAG2*	7	151406005	T	C	0.30	0.03	0.04	3.6E-01
rs17786744	*STC1*	8	23777006	G	A	0.43	0.02	0.03	5.1E-01
rs2941484	*HNF4G*	8	76478768	T	C	0.44	0.05	0.03	1.4E-01
rs10821905	*A1CF*	10	52646093	A	G	0.16	0.08	0.04	8.4E-02
rs653178	*ATXN2*	12	112007756	T	C	0.48	-0.05	0.03	1.5E-01
rs1394125	*UBE2Q2*	15	76158983	A	G	0.36	-0.04	0.04	2.5E-01
rs6598541	*IGF1R*	15	99271135	G	A	0.61	-0.03	0.03	3.3E-01
rs7193778	*NFAT5*	16	69563890	T	C	0.87	0.07	0.05	1.6E-01
rs7188445	*MAF*	16	79734987	A	G	0.34	-0.02	0.04	5.3E-01
rs7224610	*HLF*	17	53364788	A	C	0.58	-0.05	0.03	1.6E-01

SNP: previously reported SNP; Chr: chromosome; A1: coded allele; A2: non-coded allele; Freq. A1: frequency coded allele; Effects: effect of the coded allele; SE: standard error. Adjusted for age, sex, log(eGFR), BMI, significant principal components ($p<0.05$), intake of diuretics, and gout medication. P-value bold: significant p-value after adjusting for multiple testing ($p<1.9E-03$).

Supplemental Table 4: Effect size comparison of previously reported SNPs discovered among ~ 140,000 individuals from the GUGC consortium and serum urate with genome-wide significant SNPs in the GCKD study cohort (N=4941).

							**GUGC**			**serum urate**			**P-value for difference**
**SNP**	**Closest gene**	**Chr**	**Position**	**A1**	**A2**	**Freq.A1**	**effects**	**SE**	**P-value**	**effects**	**SE**	**P-value**	**GUGC/serum urate**
rs12498742	*SLC2A9*	4	9944052	G	A	0.23	-0.37	0.006	<1.0E-700	-0.28	0.04	1.9E-12	2.7E-02
rs2231142	*ABCG2*	4	89052323	T	G	0.11	0.22	0.009	1.0E-134	0.40	0.05	8.8E-14	1.5E-03

SNP: reported SNP; Chr: chromosome; A1: coded allele; A2: non-coded allele; Freq.A1: frequency coded allele; Effect: effect coded allele; SE: standard error. Adjusted for age, sex, log(eGFR), BMI, significant principal components (p<0.05), intake of diuretics, and gout medication. P-value for difference: derived from a two-sample t-test for differences in mean effect sizes between GUGC and GCKD for urate, significance threshold p<1.9E-03.

Supplemental Table 5: Association between genome-wide significant SNPs and serum urate across different categories of eGFR

kidney function	N	*ABCG2* rs2231142 (T)			*SLC2A9* rs12498742 (G)		
		effect	SE	p-value	effect	SE	p-value
eGFR <30	435	0.61	0.20	2.2E-03	0.00	0.15	9.8E-01
eGFR 30-45	1789	0.46	0.09	1.8E-07	-0.42	0.07	3.4E-10
eGFR 45-60	1646	0.27	0.09	4.6E-03	-0.18	0.07	1.0E-02
eGFR >=60	1071	0.43	0.10	2.6E-05	-0.33	0.07	8.1E-06
SNP×eGFR categories	4941	0.03	0.06	6.6E-01	0.04	0.04	3.9E-01

SE: standard error. Associations were adjusted for age, sex, BMI, intake of diuretics and gout medication, with dataset stratified by eGFR categories.

Supplemental Table 6: Detailed gene-medication interactions for selected SNPs and allopurinol, thiazides, loop diuretics, potassium sparing diuretics or losartan

SNP	medication	with this medication				without this medication				all (SNP×medication)			
		effects	SE	P-value	n	effects	SE	P-value	n	effects	SE	P-value	n
SLC2A9 rs12498742 (G)	allopurinol	-0.28	0.07	1.7E-04	1533	-0.27	0.05	7.4E-09	3408	-0.06	0.09	4.9E-01	4941
	thiazide	-0.23	0.08	2.7E-03	1570	-0.33	0.05	7.2E-13	3371	0.11	0.08	2.0E-01	4941
	loop diuretic	-0.37	0.07	1.0E-07	1900	-0.25	0.05	1.5E-07	3041	-0.08	0.08	2.9E-01	4941
	potassium sparing diuretic	-0.25	0.13	5.3E-02	489	-0.30	0.04	4.2E-13	4452	0.04	0.13	7.3E-01	4941
	losartan	-0.15	0.18	4.1E-01	189	-0.29	0.04	2.5E-12	4752	0.14	0.21	5.1E-01	4941
ABCG2 rs2231142 (T)	allopurinol	0.48	0.08	1.6E-08	1533	0.35	0.07	2.2E-07	3408	0.17	0.11	1.1E-01	4941
	thiazide	0.37	0.10	3.1E-04	1570	0.43	0.06	3.0E-12	3371	-0.06	0.11	6.0E-01	4941
	loop diuretic	0.58	0.10	2.1E-09	1900	0.30	0.06	9.3E-07	3041	0.22	0.11	4.4E-02	4941
	potassium sparing diuretic	0.59	0.18	7.8E-04	489	0.39	0.06	1.2E-12	4452	0.17	0.17	3.2E-01	4941
	losartan	0.77	0.26	3.2E-03	189	0.39	0.05	1.5E-12	4752	0.40	0.30	1.8E-01	4941
SLC17A1 rs1165151 (G)	allopurinol	0.06	0.06	3.0E-01	1533	0.06	0.04	1.4E-01	3408	0.02	0.07	8.1E-01	4941
	thiazide	0.14	0.06	2.5E-02	1570	0.03	0.04	4.4E-01	3371	0.10	0.07	1.5E-01	4941
	loop diuretic	-0.03	0.06	6.2E-01	1900	0.11	0.04	3.1E-03	3041	-0.16	0.07	2.0E-02	4941
	potassium sparing diuretic	-0.02	0.11	8.3E-01	489	0.07	0.03	3.0E-02	4452	-0.09	0.11	4.0E-01	4941
	losartan	0.10	0.15	4.9E-01	189	0.06	0.03	5.8E-02	4752	0.06	0.17	7.4E-01	4941
NRXN2 /SLC22A12 rs478607 (A)	allopurinol	-0.12	0.08	1.4E-01	1533	-0.12	0.05	2.5E-02	3408	-0.03	0.10	7.3E-01	4941
	thiazide	-0.13	0.09	1.5E-01	1570	-0.12	0.05	2.1E-02	3371	-0.02	0.10	8.7E-01	4941
	loop diuretic	-0.29	0.08	4.5E-04	1900	-0.01	0.05	7.9E-01	3041	-0.27	0.09	3.6E-03	4941
	potassium sparing diuretic	-0.11	0.16	4.9E-01	489	-0.13	0.05	6.2E-03	4452	0.04	0.16	8.1E-01	4941
	losartan	-0.30	0.23	1.8E-01	189	-0.13	0.05	6.7E-03	4752	-0.13	0.26	6.2E-01	4941

SNP	medication	only this medication				no medication**				all (SNP×medication)			
		effects	SE	P-value	n	effects	SE	P-value	n	effects	SE	P-value	n
SLC2A9 rs12498742 (G)	allopurinol	-0.23	0.15	1.4E-01	322	-0.29	0.06	3.9E-06	1503	0.00	0.16	9.9E-01	1825
	thiazide	-0.06	0.12	5.9E-01	604	-0.29	0.06	3.9E-06	1503	0.23	0.12	6.3E-02	2107
	loop diuretic	-0.44	0.11	6.3E-05	589	-0.29	0.06	3.9E-06	1503	-0.17	0.12	1.6E-01	2092
	potassium sparing diuretic	-0.48	0.30	1.1E-01	61	-0.29	0.06	3.9E-06	1503	-0.10	0.30	7.4E-01	1564
	losartan	-0.06	0.28	8.3E-01	60	-0.29	0.06	3.9E-06	1503	0.27	0.33	4.1E-01	1563
ABCG2 rs2231142 (T)	allopurinol	0.21	0.15	1.7E-01	322	0.27	0.09	1.9E-03	1503	-0.07	0.17	6.8E-01	1825
	thiazide	0.35	0.16	3.3E-02	604	0.27	0.09	1.9E-03	1503	0.11	0.17	5.1E-01	2107
	loop diuretic	0.66	0.17	1.6E-04	589	0.27	0.09	1.9E-03	1503	0.40	0.18	2.4E-02	2092
	potassium sparing diuretic	0.30	0.50	5.5E-01	61	0.27	0.09	1.9E-03	1503	-0.01	0.50	9.9E-01	1564
	losartan	0.76	0.46	1.0E-01	60	0.27	0.09	1.9E-03	1503	0.49	0.52	3.5E-01	1563
SLC17A1 rs1165151 (G)	allopurinol	0.18	0.12	1.2E-01	322	0.02	0.05	6.7E-01	1503	0.19	0.13	1.4E-01	1825
	thiazide	0.24	0.10	2.1E-02	604	0.02	0.05	6.7E-01	1503	0.21	0.11	4.6E-02	2107
	loop diuretic	-0.02	0.10	8.8E-01	589	0.02	0.05	6.7E-01	1503	-0.06	0.10	5.9E-01	2092
	potassium sparing diuretic	0.00	0.25	9.9E-01	61	0.02	0.05	6.7E-01	1503	0.02	0.25	9.4E-01	1564
	losartan	0.10	0.22	6.4E-01	60	0.02	0.05	6.7E-01	1503	0.06	0.26	8.1E-01	1563
NRXN2 /SLC22A12 rs478607 (A)	allopurinol	-0.06	0.17	7.0E-01	322	-0.03	0.07	6.9E-01	1503	-0.02	0.18	8.9E-01	1825
	thiazide	0.04	0.14	7.5E-01	604	-0.03	0.07	6.9E-01	1503	0.08	0.14	5.8E-01	2107
	loop diuretic	-0.23	0.13	9.0E-02	589	-0.03	0.07	6.9E-01	1503	-0.21	0.14	1.4E-01	2092
	potassium sparing diuretic	0.23	0.44	6.1E-01	61	-0.03	0.07	6.9E-01	1503	0.12	0.42	7.8E-01	1564
	losartan	-0.76	0.37	4.3E-02	60	-0.03	0.07	6.9E-01	1503	-0.47	0.45	3.0E-01	1563

** No medication refers to not taking gout medications, diuretics and losartan. Associations were adjusted for age, sex, log(eGFR), BMI, and medications (gout medication, diuretics and losartan) other than the category being evaluated. Multiple-testing adjusted statistical significance threshold: $p<2.5E-3$ (0.05/4*5).
